# Verrucous gastritis-like lesion in intramucosal *Helicobacter pylori*-uninfected signet ring cell carcinoma with poorly differentiated adenocarcinoma

**DOI:** 10.1007/s12328-024-01952-9

**Published:** 2024-03-23

**Authors:** Hiroki Takemoto, Takahiro Kotachi, Hajime Teshima, Hirosato Tamari, Akiyoshi Tsuboi, Hidenori Tanaka, Ken Yamashita, Yuji Urabe, Akira Ishikawa, Shiro Oka

**Affiliations:** 1 Department of Gastroenterology, Hiroshima University Hospital, 1-2-3 Kasumi, Minamiku, Hiroshima, 734-8553 Japan; 2 Department of Gastrointestinal Endoscopy and Medicine, Hiroshima University Hospital, Hiroshima, Japan; 3 Department of Molecular Pathology, Graduate School of Biomedical and Health Sciences, Hiroshima University, Hiroshima, Japan

**Keywords:** *Helicobacter pylori* uninfected gastric cancer, Signet ring cell carcinoma, Poorly differentiated adenocarcinoma, Fibromuscular obliteration, Intramucosal SRCC with PDA

## Abstract

In Japan, accessible *Helicobacter pylori* (Hp) eradication therapy is associated with an increase in the prevalence of gastric cancers (GCs) in Hp uninfected stomachs. Signet ring cell carcinoma (SRCC) is the most common of these GCs. Intramucosal SRCC with poorly differentiated adenocarcinoma (PDA) occurring in Hp uninfected gastric mucosa is rare; furthermore, many Hp uninfected pure SRCCs exhibit discoloration and flat or slightly depressed lesions, and morphological elevation is relatively rare. We report a case of intramucosal SRCC with PDA with an elevated, verrucous gastritis-like lesion in a 57-year-old male patient. In the present case, the PDA area showed dense tumor cell growth and coexisting desmoplastic and fibrotic reactions. Histopathology and immunohistochemical staining identified extensive fibromuscular obliteration with smooth muscle bundles extending from the muscularis mucosa into the lamina propria. The patient underwent curative endoscopic submucosal dissection. The reporting and analysis of such rare cases may lead to a better understanding of the characteristics of advanced Hp uninfected GCs.

## Introduction

The World Health Organization’s International Agency for Research on Cancer has classified *Helicobacter pylori* (Hp) as a carcinogen that causes gastric cancer (GC) [1]. National health insurance coverage for Hp eradication therapy for the treatment of Hp associated chronic gastritis has become available in Japan. As a result, the number of patients with reduced Hp infected status and the relative number of cases of Hp uninfected GC has increased. The frequency of Hp uninfected GC ranges from 0.4 to 5.0% [2–7]. Among these cases, signet ring cell carcinoma (SRCC), characterized by a discolored and flat or slightly depressed lesion, accounts for over 90% of cases [8]. SRCC in Hp uninfected gastric mucosa shows lower proliferative activity, lesser extensive spread, and slower progression than SRCC in Hp infected gastric mucosa [4]. However, although extremely rare, cases of advanced Hp uninfected GC have also been reported [9, 10]. The invasive potential of SRCC increases after *TP53* mutation, and the cancer progresses to poorly differentiated adenocarcinoma (PDA) [11]. We report a case involving a small, flat-elevated, depressed SRCC lesion with PDA occurring in Hp uninfected gastric mucosa that was curatively resected by endoscopic submucosal dissection (ESD).

## Case report

**Fig. 1:**
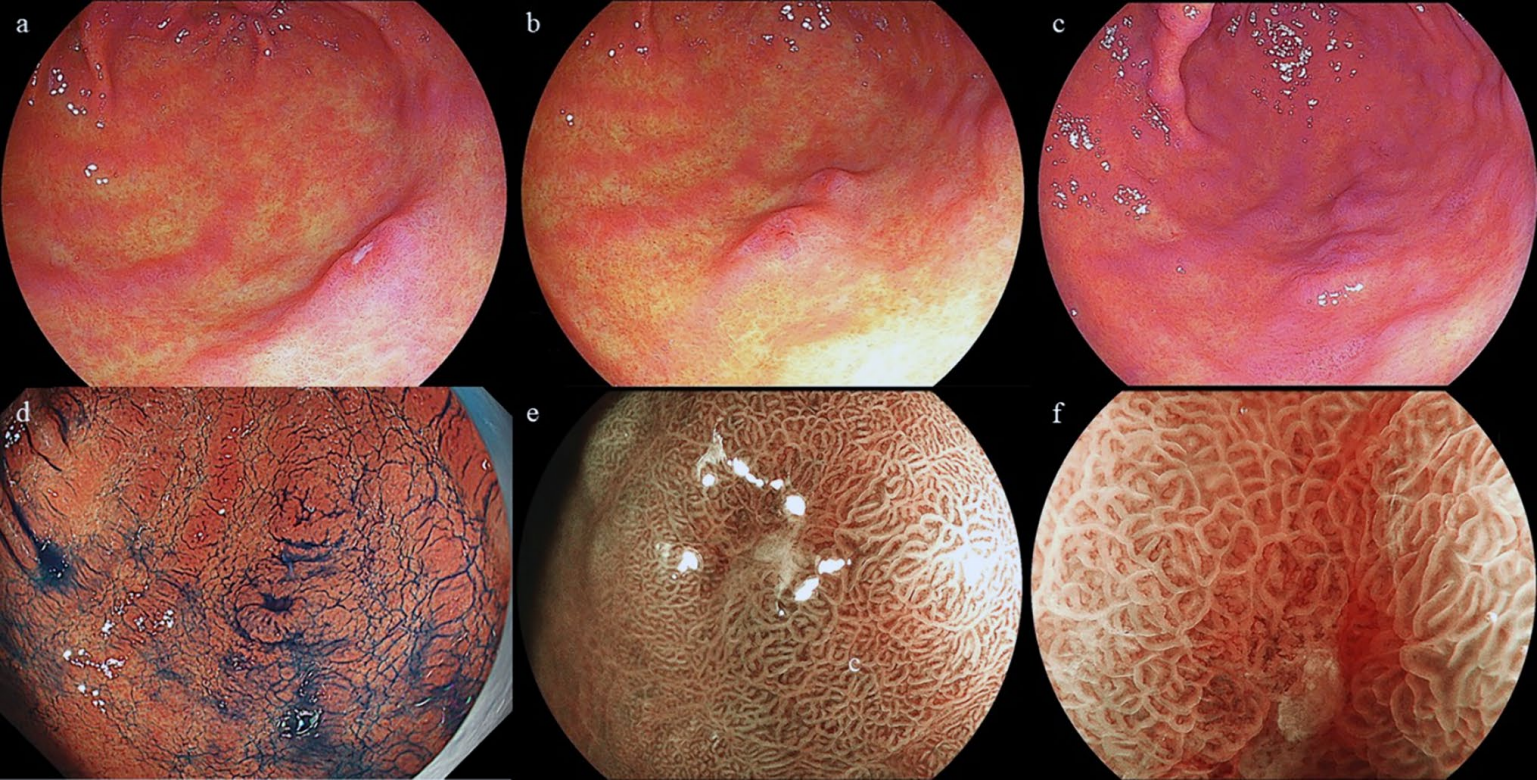
**a** An 8 mm poorly demarcated reddish elevated verrucous gastritis-like lesion under linked color imaging (LCI) located at the greater curvature of the gastric antrum. **b** This lesion changed to a two-hump shape after 1 year. **c** Although this lesion was slightly lower in height than what was observed in the previous EGD, the findings were similar. **d** After the indigo carmine dye spraying was applied, no encroachment was observed on the fold. **e, f** Unclear demarcation line under blue laser imaging (BLI) that was partially covered with normal mucosa. The lesion was elevated with a swollen glandular structure and a slightly depressed area in the dilated intervening part were observed

The patient was a 57-year-old male. He had no symptoms and was diagnosed with early GC. A depressed lesion of 10 mm in diameter located at the greater curvature of the gastric antrum was found during a routine upper gastrointestinal endoscopy procedure. He was referred to Hiroshima University Hospital, Hiroshima, Japan, after a biopsy indicating SRCC. The patient had type 2 diabetes mellitus, dyslipidemia, and postoperative renal cell carcinoma. His family history included rectal cancer in his mother and no history of GC. He smoked 15 cigarettes (Brinkman index = 600) and drank 60 mL of whiskey per day for 40 years. The patient had no history of Hp eradication therapy. Hp stool antigen, urea breath, and serum immunoglobulin G antibody tests were negative. An EGD showed a regular arrangement of collecting venules (RAC) [12] in the entire gastric fundic gland area and no atrophic mucosal change. The histopathological results of five biopsies previously performed at our hospital did not show mucosal atrophy. From the above laboratory data, endoscopic findings, and histopathological findings, we concluded that the stomach had a normal gastric mucosa without Hp infection. The patient underwent EGD annually until 3 years before being referred to our hospital. One year before being referred to our hospital, an EGD revealed an 8 mm poorly demarcated, reddish, elevated, verrucous gastritis-like lesion under linked color imaging (LCI). This lesion was located at the greater curvature of the gastric antrum and was a single lesion with erosion at the top (Fig. 1a). No biopsy was performed in this EGD. One month before being referred to our hospital, an EGD revealed that the lesion’s appearance was similar to that of 1 year ago. However, the lesion changed to a two-hump shape (Fig. 1b). Biopsy was performed from a lesion on the oral side and indicated an SRCC. Compared to the previous EGD, the lesion was slightly smaller in height, revealing a 10 mm poorly demarcated elevated verrucous gastritis-like lesion with a slight depression at the top (Fig. 1c). Application of indigo carmine sprinkling solution clearly showed the depressed area. However, this lesion did not show encroachment of the fold (Fig. 1d). Moreover, the lesion had an unclear demarcation line (BLI) under blue laser imaging and was partially covered with normal mucosa (Fig. 1e). The lesion was elevated with a swollen glandular structure, and a slightly depressed area in the dilated intervening part was observed (Fig. 1e, f). We assumed this lesion to be a malignant tumor with 0–IIa+IIc according to the Paris classification. The lesion was small, neither thickened nor hard, and was diagnosed as intramucosal carcinoma. And negative biopsy results were collected from four sites surrounding the lesion (5–10 mm from the tumor margin). Therefore, we performed ESD.

**Fig. 2:**
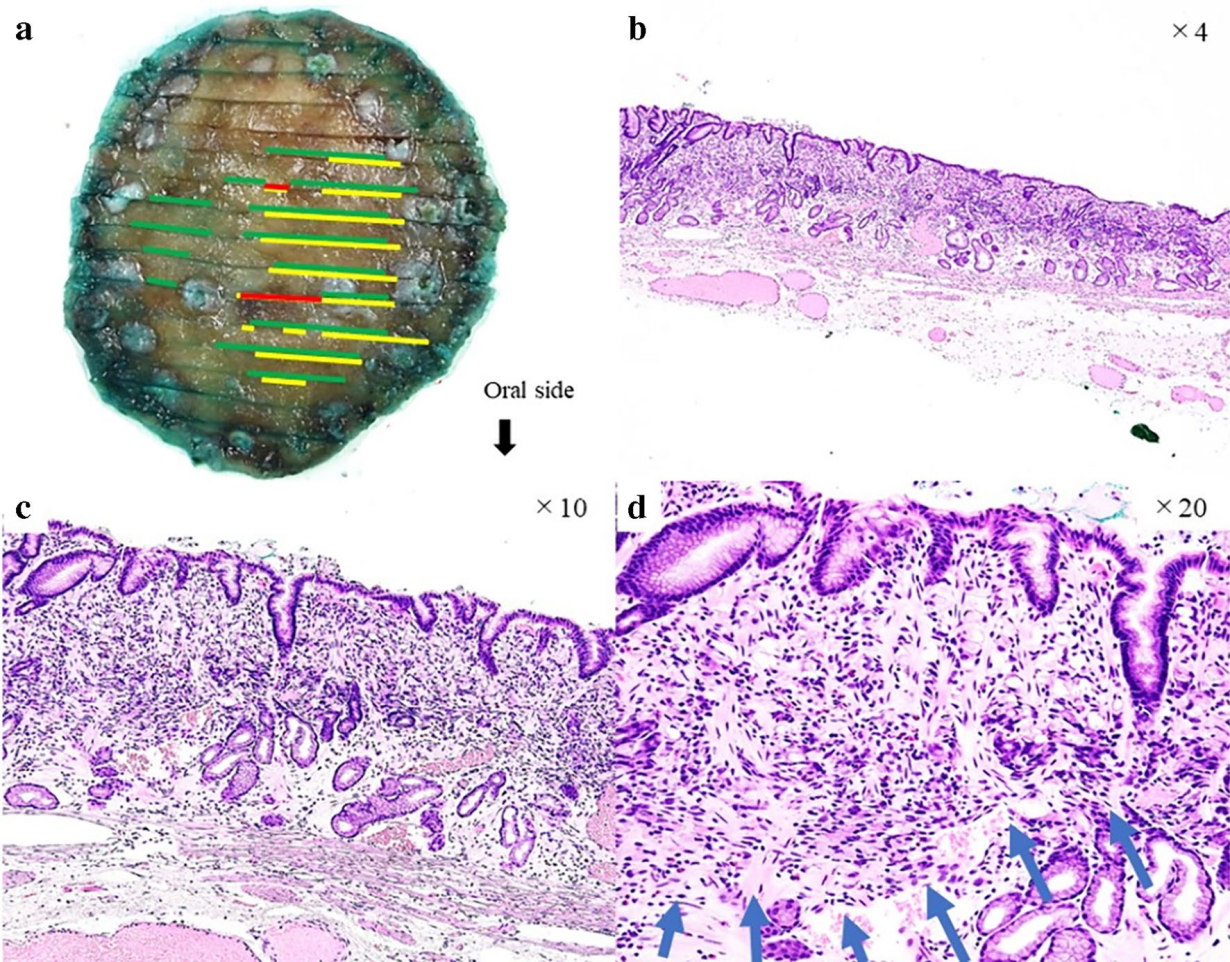
**a** Histopathological mapping of the resected specimen. The yellow line indicates signet ring cell carcinoma (SRCC). The red line indicates SRCC with poorly differentiated adenocarcinoma (PDA). The green line indicates fibromuscular obliteration strong positive. **b** Hematoxylin and Eosin staining showed that the SRCC was in the lamina propria layer and grew into the lumen forming an elevated lesion that was covered by normal mucosa. **c, d** The slightly depressed area at the top of the lesion contained a higher density of tumor cells compared with the surrounding area and was associated with PDA component, which was also covered by normal mucosa. The blue arrow indicates dense cancer cell growth and coexistent desmoplastic and fibrotic reactions

**Fig. 3:**
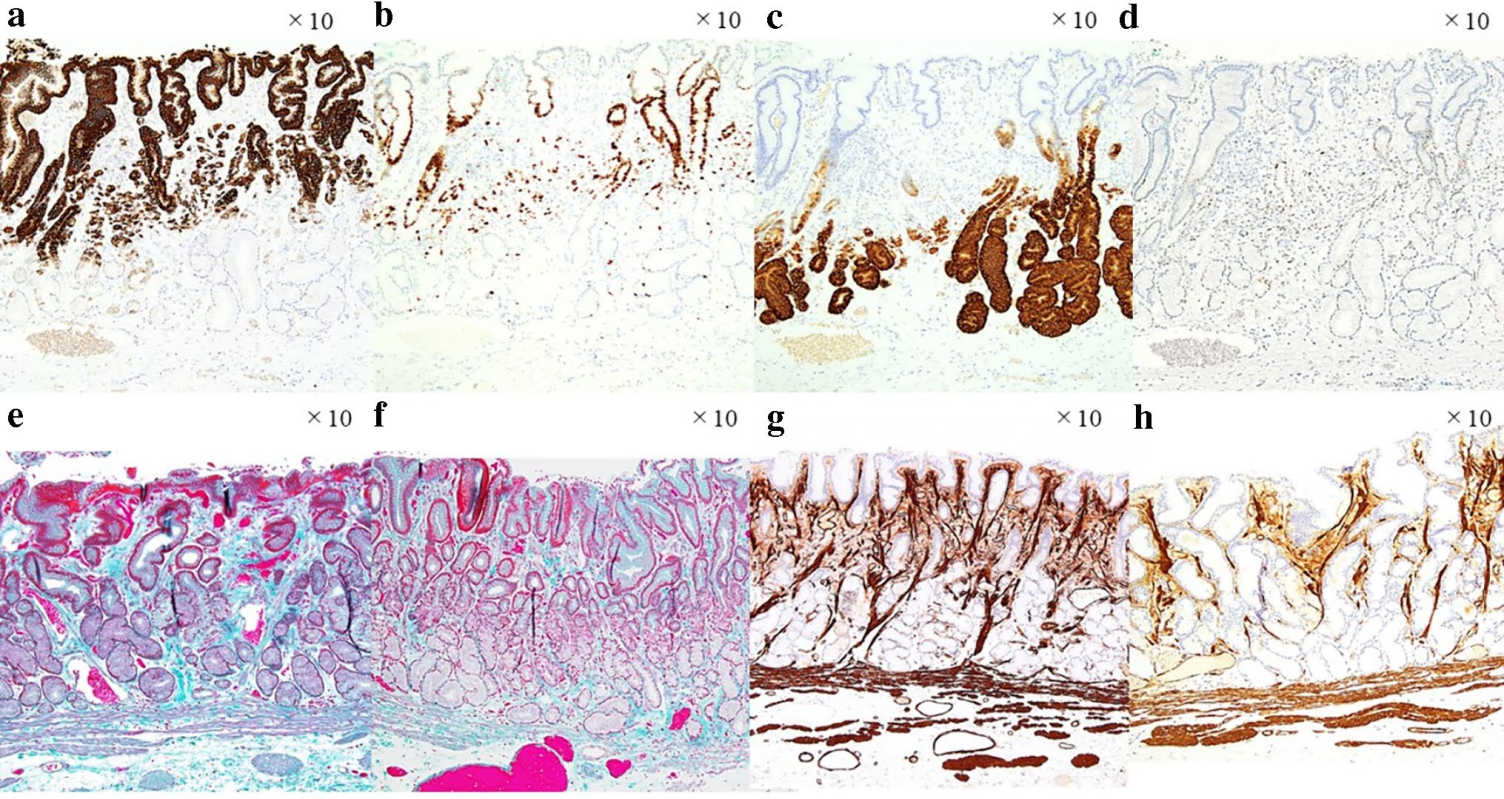
**a** The SRCC area and foveolar epithelium, spread over the superficial layer, were MUC5AC-positive. **b** The deep PDA area, extending to the middle intramucosal layer, was Ki-67-positive. **c** A part of the PDA area and pyloric glands and mucous neck cells, spread in the deep layer, were MUC6-positive. **d** A part of the PDA area was p53 positive; however, very few SRCC cells were p53-positive. **e** Masson trichrome staining revealed extensive fibromuscular obliteration. **f** This area was weakly fibromuscular as shown by obliteration in the Masson trichrome staining. **g** Anti-α-smooth muscle actin staining revealed fibromuscular obliteration. **h** This area was weakly fibromuscular as shown by obliteration in anti-α-smooth muscle actin staining

The resected specimen measured 35×30 mm, and the lesion measured 18×16 mm. Figure 2a shows the histopathological mapping of the resected specimen, wherein the yellow and red lines indicated pure SRCC and SRCC with PDA, respectively. The green line indicates fibromuscular obliteration strong positive. Hematoxylin and Eosin (HE) staining showed that the SRCC was localized to the lamina propria and covered by non-neoplastic foveolar epithelium, and the SRCC developed toward the luminal side, forming a lesion that was taller than the surrounding non-neoplastic mucosa. (Fig. 2b). In addition, the slightly depressed area at the top of the lesion contained a higher density of tumor cells compared with the surrounding area and was associated with the PDA component, which was also covered by non-neoplastic foveolar epithelium (Fig. 2c, d). In Fig. 2d, the blue arrow indicated dense cancer cell growth and coexistent desmoplastic and fibrotic reactions. No erosions or tumor cell exposure on the surface was observed. Immunohistochemical staining showed that tumor cells could be divided into two layers, superficial and deep. The SRCC area and foveolar epithelium, spread over the superficial layer, were MUC5AC-positive (Fig. 3a). The deep PDA area, extending to the middle intramucosal layer, was Ki-67-positive (Fig. 3b). The part of PDA area and pyloric glands and mucous neck cells, spread in the deep layer, were MUC6-positive (Fig. 3c). This layered structure was similar to that of the normal gastric mucosa. In addition, the deep PDA cells were positive for Ki-67 (Fig. 3b) and p53 (Fig. 3d); however, very few SRCC cells were Ki-67- and p53-positive. Both components were negative for MUC2 and CD10. Particularly, the p53-positive PDA cell rate was more than 50%. In the background non-neoplastic mucosa surrounding the lesion and the cancer lesion, Masson trichrome (Fig. 3e) and anti-α-smooth muscle actin (Fig. 3g) staining revealed that extensive fibromuscular obliteration was characterized by dense, proliferative, thin muscle bundles, which extended from the muscularis mucosa to the lamina propria; however, some areas were weakly positive (Fig. 3f, h). In the ESD specimen, the surrounding mucosa was almost entirely composed of pyloric glands. Thus, this lesion was considered an intramucosal SRCC concomitant with PDA, had clear resection margins, and had no lymphovascular invasion. Resection was considered curative. Additional surgical resection was not needed, the patient chose to undergo annual endoscopic surveillance.

## Discussion

This case had two unique features. First, SRCC with PDA was detected. Hp uninfected undifferentiated-type GC (UD-GC) is present in most SRCC cases [3, 5, 13]. In particular, cases of pure Hp uninfected SRCC exhibit intramucosal lesions [8]. Many cases of advanced GC, including Hp exposed UD-GC, exhibit PDA. In a mouse model, p53 loss promotes cell growth and anti-apoptotic events [9] and is associated with the progression of intramucosal UD-GC to advanced UD-GC. Therefore, intramucosal carcinoma cells are SRCC but the invasive area changes to PDA, and mutations, such as p53 loss, likely result in increased invasive potential and morphological changes [14]. In this patient, immunohistochemical staining for p53 in the PDA area was positive. PDA can invade the submucosa, and curative resection of intramucosal carcinoma can be achieved via early identification and treatment of the lesion. In addition, the layered structure was similar to that of normal gastric glands. In the intramucosal layer, SRCC often exhibits a double-layer structure (DLS) [15]. Natsagdorj et al. [16]. reported that the presence of the DLS markedly decreased with submucosal invasion. The lesion in our case exhibited a DLS but the increasing PDA area size may have compromised the maintenance of the DLS. Very few cases of Hp uninfected advanced GC have been reported, and histopathological examination in these cases shows either SRCC, SRCC with PDA, or pure PDA [17]. The other important feature of this lesion was elevation. Many Hp uninfected pure SRCCs exhibit discoloration and flat or slightly depressed lesions [8]. However, only a few cases exhibit elevated lesions [18–20]. Kishi et al. [18]. reported that the mechanism underlying the protrusion of UD-GC lesions has not been determined but four possibilities have been proposed: formation of fibrous tissue or mucinous nodules due to cancer invasion into the deep layer of the gastric mucosa, dense growth of cancer cells, proliferation of UD-GC in a gastric hyperplastic polyp, or fibromuscular obliteration of the muscularis propria. We think that the elevated lesion occurred due to two factors. The PDA area showed dense cancer cell growth and coexistent desmoplastic and fibrotic reactions, which may have caused the elevation of the lesion. The lesion was located in the gastric antrum, and fibromuscular obliteration of the muscularis propria caused an elevated lesion. Fibromuscular obliteration of the muscularis propria is reported in conditions such as rectal mucosal prolapse syndrome and occurs when smooth muscle bundles extend from the muscularis mucosa into the lamina propria [21]. In our case, immunohistochemical staining indicated fibromuscular obliteration around the pyloric glands in the background mucosa surrounding the lesion.

Among Hp uninfected GCs, intestinal-type differentiated carcinoma is known as a typical type of Hp uninfected GC arising in the pyloric gland region and is similar to verrucous gastritis-like lesion. This patient was referred to our hospital after a biopsy indicated SRCC. It thus remains to be discussed how this case can be differentiated from intestinal-type differentiated carcinoma or verrucous gastritis (VG). Intestinal-type differentiated carcinoma in Hp uninfected GC has been reported to show flat, elevated, or depressed lesions [22] and a single erosion [23]. Irregular microvascular patterns on magnifying endoscopy with narrow-band imaging (ME-NBI) can help distinguish differentiated tubular adenocarcinoma from the surrounding erosions [24, 25], while other ME-NBI findings have reported an absence of a clear DL or an irregular microvessel/surface pattern [23]. Moreover, in VG, the lesions are arranged along longitudinal folds converging toward the pylorus ring. Tsuji et al. [26] reported that Hp negativity was independently associated with VG regardless of the history of Hp eradication. Moreover, when endoscopic findings show flat elevated or depressed lesions, the differential diagnosis should include VG or early GC. In the present case, the lesion was partially covered with normal mucosa and did not show irregular microvascular pattern under BLI. Furthermore, elevated intramucosal UD-GC in Hp uninfected cases are rare case. A few cases [18–20, 27], wherein the lesions were macroscopically divided into protruding or submucosal tumor-like (SMT-like). And SMT-like lesions were covered with normal mucosa, did not show a clear DL or an irregular microvessel/ surface pattern on ME-NBI [18–20]. From the above findings it is clear that in many cases similar endoscopic findings are present, and although some cases can be differentiated by ME-NBI, this is not possible in all cases. In addition, elevated intramucosal UD-GCs in Hp uninfected cases are so rare that it is not well known; hence, it is difficult to differentiate the lesion from intestinal-type differentiated carcinoma and VG. Therefore, when a single lesion is observed in the antrum, a biopsy of the lesion should be performed. Additional, in this case, endoscopic findings of the extension were difficult by covered normal mucosa, we performed negative biopsies. In this resected specimen, tumor cells were present consistent with an elevated lesion. UD-GC incidence of positive lateral margins in en bloc specimens resected by ESD has been reported to be 27.3% [28], and previous studies reveal that the predictive factors for inaccurate determination of the lateral extent of early gastric cancer indicated for ESD include UD-GC [29, 30]. And it was reported that it would be difficult to delineate diagnostic demarcation, even using magnifying endoscopy with NBI, in cases with a narrower inter-crypt distance in the cancerous region because of the scarcity of cancer cells as well as in cases with pronounced inflammatory cell infiltration [31]. Negative biopsies help determine the lateral extent of the lesion [32–34]. Although we need to pay attention to the dilated intervening part [31, 35], elevated intramucosal UD-GCs in Hp uninfected cases are so rare, there is no sufficient consensus, we thought that need for negative biopsies in surrounding the lesion (5–10 mm from the tumor margin).

In conclusion, we reported the case of a patient with an elevated, verrucous gastritis-like lesion who underwent ESD. The histopathological diagnosis was intramucosal SRCC with PDA with negative lymphovascular invasion and clear resection margins. The resection was curative. This report may improve our understanding of the characteristics of rare, advanced Hp uninfected GCs.

## References

[1] IARC Working Group on the Evaluation of Carcinogenic Risks to Humans. Schistosomes, liver flukes and *Helicobacter pylori* IARC. Monogr Eval Carcinog Risks Hum 1994;61:241 PMC7681621 7715068

[2] Ono S, Kato M, Suzuki M (2012). Frequency of *Helicobacter pylori*-negative gastric cancer and gastric mucosal atrophy in a Japanese endoscopic submucosal dissection series including histological, endoscopic and serological atrophy. Digestion.

[3] Matsuo T, Ito M, Takata S (2011). Low prevalence of *Helicobacter pylori*-negative gastric cancer among Japanese. Helicobacter.

[4] Horiuchi Y, Fujisaki J, Yamamoto N (2016). Biological behavior of the intramucosal *Helicobacter pylori*-negative undifferentiated-type early gastric cancer: comparison with *Helicobacter pylori*-positive early gastric cancer. Gastric Cancer.

[5] Kato S, Matsukura N, Tsukada K (2007). *Helicobacter pylori* infection-negative gastric cancer in Japanese hospital patients: incidence and pathological characteristics. Cancer Sci.

[6] Yoon H, Kim N, Lee HS (2011). *Helicobacter pylori*-negative gastric cancer in South Korea: incidence and clinicopathologic characteristics. Helicobacter.

[7] Yamada A, Kaise M, Inoshita N (2018). Characterization of *Helicobacter pylori* uninfected early gastric cancers. Digestion.

[8] Yamamoto Y, Fujisaki J, Omae M (2015). *Helicobacter pylori*-negative gastric cancer: characteristics and endoscopic findings. Dig Endosc.

[9] Okano A, Kato S, Ohana M (2017). *Helicobacter pylori*-negative gastric cancer: advanced-stage undifferentiated adenocarcinoma located in the pyloric gland area. Clin J Gastroenterol.

[10] Takagi A, Ozawa H, Oki M (2018). *Helicobacter pylori*-negative advanced gastric cancer with massive eosinophilia. Intern Med.

[11] Shimada S, Mimata A, Sekine M (2012). Synergistic tumour suppressor activity of E-cadherin and p53 in a conditional mouse model for metastatic diffuse-type gastric cancer. Gut.

[12] Yagi K, Aruga Y, Nakamura A (2005). Regular arrangement of collecting venules (RAC): a characteristic endoscopic feature of *Helicobacter pylori*-negative normal stomach and its relationship with esophago-gastric adenocarcinoma. J Gastroenterol.

[13] Kakinoki R, Kushima R, Matsubara A (2009). Re-evaluation of histogenesis of gastric carcinomas: a comparative histopathological study between *Helicobacter pylori*-negative and *H. pylori*-positive cases. Dig Dis Sci.

[14] Kiso M, Urabe Y, Ito M (2020). Clinical and genomic characteristics of mucosal signet-ring cell carcinoma in *Helicobacter pylori*-uninfected stomach. BMC Gastroenterol.

[15] Sugihara H, Hattori T, Fukuda M (1987). Cell proliferation and differentiation in intramucosal and advanced signet ring cell carcinomas of the human stomach. Virchows Arch A Pathol Anat Histopathol.

[16] Natsagdorj L, Sugihara H, Bamba M (2008). Intratumoural heterogeneity of intestinal expression reflects environmental induction and progression-related loss of induction in undifferentiated-type gastric carcinomas. Histopathology.

[17] Yoshimura D, Yoshimura R, Kato S (2020). Clinical and pathological characteristics of *Helicobacter pylori*-uninfected signet-ring cell cancer of the stomach an analysis of case series. Stomach Intestine.

[18] Kishi K, Adachi K, Sakamoto U (2023). Gastric flat elevated undifferentiated signet-ring cell mucosal cancer lesion detected in a case without *Helicobacter pylori* infection. Intern Med.

[19] Misumi Y, Ichihara S, Nonaka K (2021). Gastric signet-ring cell carcinoma that presented as an elevated lesion due to fibromuscular obliteration in the lamina propria. Case rep Gastrointest.

[20] Shiratori Y, Ikeya T, Suzuki K (2020). The rare case of elevated signet ring cell gastric carcinoma with *Helicobacter pylori* uninfected mucosa. Clin J Gastroenterol.

[21] Murata M, Sugimoto M, Ban H (2017). Cap polyposis refractory to *Helicobacter pylori* eradication treated with endoscopic submucosal dissection. World J Gastrointest Endosc.

[22] Murawaki Y, Yashima K, Horie S (2023). Helicobacter pylori-negative gastric adenocarcinoma mimicking VG in the antrum: a case report and literature review. Intern Med.

[23] Takita M, Ohata K, Inamoto R (2021). Endoscopic and histological feature of *Helicobacter pylor*i-negative differentiated gastric adenocarcinoma arising in antrum. JGH Open.

[24] Kotani S, Miyaoka Y, Fujiwara A (2016). Intestinal-type gastric adenocarcinoma without *Helicobacter pylori* infection successfully treated with endoscopic submucosal dissection. Clin J Gastroenterol.

[25] Ozaki Y, Suto H, Nasaka T (2015). A case of *Helicobacter pylori*-negative intramucosal well-differentiated gastric adenocarcinoma with intestinal phenotype. Clin J Gastroenterol.

[26] Tsuji N, Umehara Y, Takenaka M (2019). Verrucous antral gastritis in relation to *Helicobacter pylori* infection, nutrition, and gastric atrophy. Gastroenterol Rep.

[27] Oura R, Katayama Y, Gyotoku Y (2016). A case of undifferentiated intramucosal gastric cancer that exhibited elevated type. Dokkyo J Med Sci.

[28] Jeon HK, Lee SJ, Kim GH (2018). Endoscopic submucosal dissection for undifferentiated-type early gastric cancer: short- and long-term outcomes. Surg Endosc.

[29] Kakushima N, Ono H, Tanaka M (2011). Factors related to lateral margin positivity for cancer in gastric specimens of endoscopic submucosal dissection. Dig Endosc.

[30] Asada-Hirayama I, Kodashima S, Goto O (2013). Factors predictive of inaccurate determination of horizontal extent of intestinal-type early gastric cancers during endoscopic submucosal dissection: a retrospective analysis. Dig Endosc.

[31] Horiuchi Y, Fujisaki J, Yamamoto N (2016). Accuracy of diagnostic demarcation of undifferentiated-type early gastric cancers for magnifying endoscopy with narrow-band imaging: endoscopic submucosal dissection cases. Gastric Cancer.

[32] Yamamoto Y, Fujisaki J, Hirasawa T (2010). Therapeutic outcomes of endoscopic submucosal dissection of undifferentiated-type intramucosal gastric cancer without ulceration and preoperatively diagnosed as 20 millimetres or less in diameter. Dig Endosc.

[33] Yoshimizu S, Yamamoto Y, Horiuchi Y (2019). A suitable marking method to achieve lateral margin negative in endoscopic submucosal dissection for undifferentiated-type early gastric cancer. Endosc Int Open.

[34] Ono H, Yao K, Fujishiro M (2021). Guidelines for endoscopic submucosal dissection and endoscopic mucosal resection for early gastric cancer (second edition). Dig Endosc.

[35] Okada K, Fujisaki J, Kasuga A (2011). Diagnosis of undifferentiated-type early gastric cancers by magnification endoscopy with narrow-band imaging. J Gastroenterol Hepatol.

